# Experimental and Numerical Investigation of Mechanical and Thermal Effects in TiNi SMA during Transformation-Induced Creep Phenomena

**DOI:** 10.3390/ma12060883

**Published:** 2019-03-16

**Authors:** Vladimir Dunić, Elżbieta A. Pieczyska, Zbigniew L. Kowalewski, Ryosuke Matsui, Radovan Slavković

**Affiliations:** 1 University of Kragujevac, Faculty of Engineering, Sestre Janjić 6, 34000 Kragujevac, Serbia; dunic@kg.ac.rs (V.D.); radovan@kg.ac.rs (R.S.); 2 Institute of Fundamental Technological Research, Polish Academy of Sciences, Pawińskiego 5b, 02-106 Warsaw, Poland; zkowalew@ippt.pan.pl; 3 Department of Mechanical Engineering, Aichi Institute of Technology, 1247 Yachigusa, Yakusa-cho, Toyota 470-0392, Japan; r_matsui@aitech.ac.jp

**Keywords:** TiNi shape memory alloy, phase transformation-induced creep, martensitic transformation, temperature change, thermomechanical couplings, infrared camera, thermo-mechanical coupled numerical analysis

## Abstract

(1) The paper presents experimental and numerical results of the TiNi shape memory alloy (SMA) subjected to a modified program of force-controlled tensile loading. The time-dependent development of transformation strain under the constant-force conditions was investigated to describe transformation-induced creep phenomena. (2) Mechanical characteristics of the TiNi SMA were derived using a testing machine, whereas the SMA temperature changes accompanying its deformation were obtained in a contactless manner with an infrared camera. A 3D coupled thermo-mechanical numerical analysis, realized in a partitioned approach, was applied to describe the SMA mechanical and thermal responses. (3) The stress and related temperature changes demonstrated how the transformation-induced creep process started and evolved at various stages of the SMA loading. The proposed model reproduced the stress, strain and temperature changes obtained during the experiment well; the latent heat production is in correlation with the amount of the martensitic volume fraction. (4) It was demonstrated how the transformation-induced creep process occurring in the SMA under such conditions was involved in thermo-mechanical couplings and the related temperature changes.

## 1. Introduction

It is of great importance to understand and to predict the behavior of materials and structures in cases which are usually encountered in contemporary applications. An interruption of the loading, due to the accidents or a shortage of energy supply, is a situation that needs to be studied to anticipate the behavior of devices made of SMA such as sensors or actuators. Recent research on this topic, e.g. Matsui et al. [1], Pieczyska et al. [2,3], considered that the strain-controlled loading interruption, when the strain was kept constant, leads to the stress relaxation in the loading branch of the stress-strain curve, and the stress increase in the unloading one. It was recognized that such behavior is related to thermomechanical coupling. Russalian and Bhattacharyya found out that the phenomena can appear during reorientation loading and the isothermal pseudo-elasticity as well [4]. They conducted a 12-h experiment and recorded that stress arrest leads to strain accumulation, mainly in the first 10 minutes. On the other hand, Grabe and Bruhns in [5] compared the isothermal and non-isothermal loadings and noted that no stress relaxation was discovered for relaxation tests conducted under the isothermal conditions. There is the remark, reported by Tobushi et al., that the observed pseudo-viscous relaxation effects are mainly caused by variation of the SMA temperature [6]. 

Pieczyska in [7] presented the procedure of experimental investigation of the force-controlled tensile loading of the TiNi SMA and studied the state and progress of the stress-induced transformation based on the temperature variation, captured by infrared camera. It was demonstrated that the nature of the SMA temperature change can manifest the current stage of the phase transformation. Takeda et al. investigated also the transformation-induced stress relaxation and recovery in the TiNi SMA [8]. They concluded that for the higher loading rates, a loading break gave higher stress variation resulting in high thermo-sensitivity of the SMA.

The general assumption is that deformations in material occur immediately after the application of the loading. The creep behavior in steel has been described as a time-dependent inelastic deformation which induces development of the permanent strains with time [9]. In the case of SMA, the inelastic deformations are mainly the result of the martensitic phase transformation. So, the occurrence of deformation in SMA while keeping the loading at a constant level can be classified as creep-like deformation.

The goal of this paper is to investigate a creep-like deformation in SMA caused by the phase transformation. To this end, the modified tensile loading program for the TiNi SMA was conducted under the loading force kept constant at various stages of the stress-induced martensitic transformation (SIMT). The thermo-mechanical coupled numerical analysis using the finite element method (FEM) was engaged and the transformation-induced creep-like phenomena were analyzed.

## 2. Experimental Details

**Figure 1:**
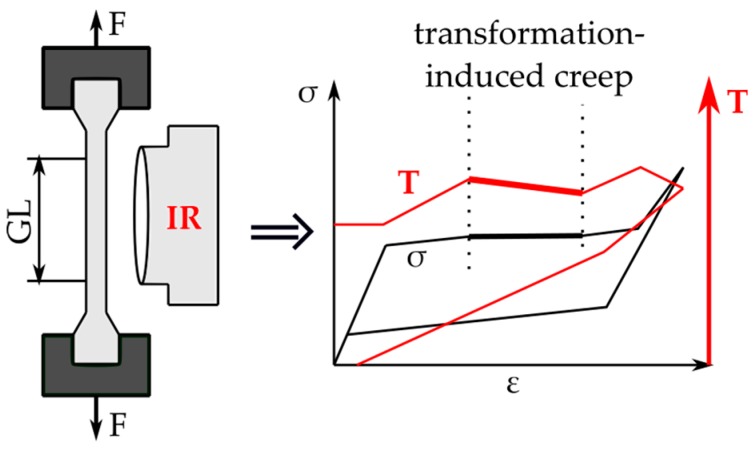
Scheme of the experimental set-up applied for transformation-induced creep-like phenomena in shape memory alloy (SMA).

The experiments were carried out on the TiNi SMA specimens produced by the Furukawa Electric Co. in Japan. The SMA constitution was Ti-55.3 wt% Ni. Its austenite finish temperature *A_f_* was approximately 283 K. The length, width and thickness of the belt type specimens were equal to 160 × 10 × 0.40 mm^3^. Each specimen was subjected to the force-controlled tensile loading at room temperature on an Instron 5867 testing machine. The estimated stress rate of the loading and unloading process was 12.5 MPa s^−1^. The fast and sensitive infrared camera (IR) Therma CAM^tm^ FLIR Co. was used to record the infrared radiation from the specimen surface during the loading. The infrared system enabled us to obtain the temperature distribution and to acquire the average temperature of the specimen in a contactless manner with high accuracy. To increase the SMA emissivity and make it uniform, the specimen surface was covered with a thin layer of the black carbon powder characterized by the coefficient of thermal emissivity circa 0.95. From the temperature distributions obtained, the mean temperature of the SMA specimen for each instant of the straining was determined with high sensitivity of 0.02 K. The scheme of the experimental setup is presented in Figure 1.

A difference between the mean value of temperature $T_{mean}(t)$ calculated for the gauge part of the specimen and the mean temperature of the same area before the deformation $T_{mean}\left(t_{0}\right)$ was calculated in order to analyze the thermal effects accompanying the SMA loading process:

(1) $$\Delta T=T_{mean}\left(t\right)-T_{mean}\left(t_{0}\right).$$

The temperature variation can be presented as a function of time, strain or stress (see Section 4).

The modified process of the SMA loading proposed in the experiment was conducted according to the three different programs (1)–(3), i.e., the loading force was kept constant approximately three minutes at various stages of the localized martensitic transformation (LMT), monitored by infrared camera):

- at the beginning of the LMT stage (thermogram 1)–Program (1);
- in the middle of the LMT stage (thermogram 2)–Program (2);
- at the end of the LMT stage (thermogram 3)–Program (3).

**Figure 2:**
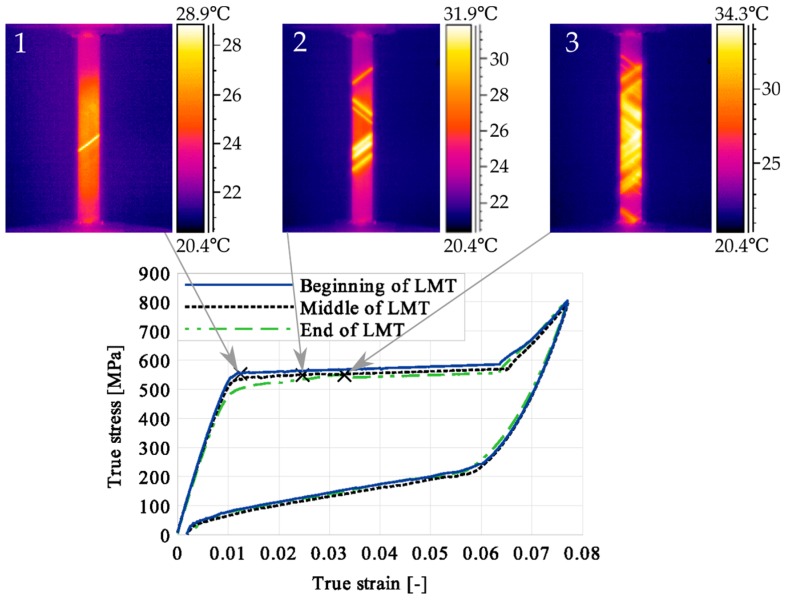
Stress–strain curve of TiNi SMA under force-controlled conditions; the marked points correspond to the thermograms 1, 2 and 3.

The pointed loading and transformation stages 1, 2 and 3 were determined by the infrared camera (Figure 2).

## 3. Thermo-Mechanical Coupling of Finite Element Method (FEM) Software

The PAKS [10] is FEM-based program for linear or nonlinear structural analysis. As the result of the iterative procedure which solves internal $F^{\mathrm{int}}$ and external forces $F^{ext}$ equilibrium [9], the displacement field at the time t+Δt is obtained by:

(2) $$K_{n}\Delta u={}^{t+\Delta t}F^{ext}-{}^{t}F^{\mathrm{int}},$$

(3) $${}^{t+\Delta t}u\approx {}^{t}u+\Delta u,$$

where $K_{n}$ is stiffness matrix, u is displacement field vector, Δu is vector of displacement increment, t is time, and Δt is time increment.

PAKT [11] is the heat-transfer analysis FEM program which enables numerical computation of a temperature change in solid bodies under the following boundary conditions: convection on the part of the surface, prescribed surface flux, prescribed temperature, and radiation. It is based on the energy balance equation expressed by the Fourier’s law of heat conduction [9]:

(4) $$-\rho c\frac{\partial T}{\partial t}+\nabla^{T}\left(k\nabla T\right)+q+\left(q_{diss}-T_{0}\alpha c_{m}{\dot{e}}_{m}\right)=0,$$

where: *ρ*-density of the material, T-absolute temperature, c-effective specific heat, q-local heat source, k-material’s conductivity, **T** = ∇*T*-temperature gradient. 

**Figure 3:**
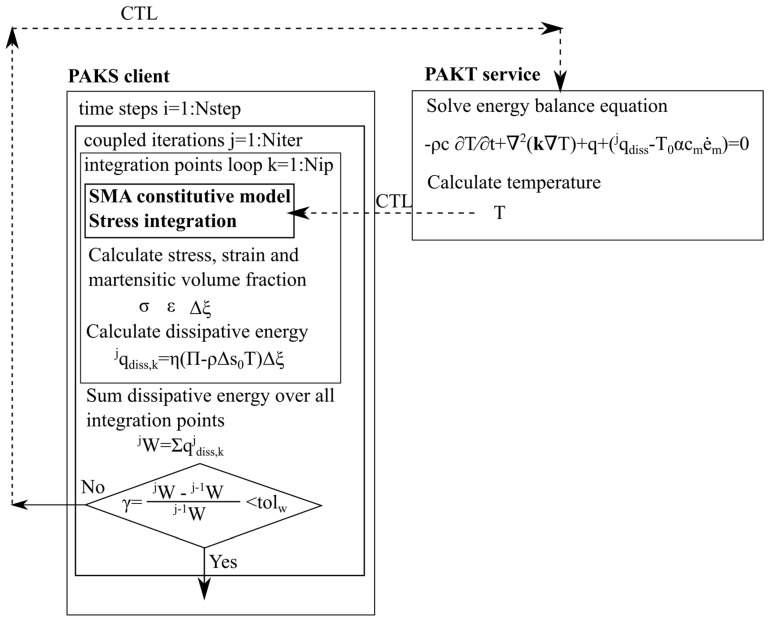
Scheme of the iterative procedure for thermo-mechanical coupling analysis.

In the last term of Equation (4), the elementary dissipative energy of martensitic transformation $q_{diss}$ and the thermo-elastic effect $-T_{0}\alpha c_{m}{\dot{e}}_{m}$ are introduced as additional heat source and sink, where $T_{0}$ is ambient temperature, α is coefficient of thermal expansion, $c_{m}$ is bulk modulus, and ${\dot{e}}_{m}$ is rate of mean strain. The elementary dissipative energy $q_{diss}$ of the martensitic phase transformation in the SMA was defined in [12] and may be expressed in the following way:

(5) $$q_{diss}=\eta \left(\Pi -\rho \Delta s_{0}T\right)\Delta \xi ,$$

where η is dissipative factor, $\rho \Delta s_{0}$ is stress sensitivity coefficient, Π is thermodynamic force, and Δξ is martensitic volume fraction increment [12]. The temperature T is computed in the PAKT. The procedure of thermo-mechanical coupling of the FEM software programs has been proposed by Matthies et al. [13]. The Component Template Library (CTL) developed by Niekamp [14] serves as the library solution providing data exchange between the structural analysis PAKS and the heat transfer analysis PAKT. The exchange of data provides a possibility to solve coupled problems [15,16]. The schematic representation of the thermo-mechanical coupling iterative procedure is given in Figure 3.

## 4. Experimental and Modeling Results

The goal of the proposed research is thermo-mechanical behavior of the TiNi SMA under the force-controlled loading conditions when a loading interruption was induced at various stages: at the initial (program 1), advanced (program 2) and almost completed stress-induced martensite transformation (program 3).

During the experiment, the SMA belt type specimens (160 × 10 × 0.40 mm^3^) were clamped on both ends by the grips of the testing machine. The loading was applied on one side, while the other side was fixed. The computer connected with the testing machine recorded the displacements for the prescribed force, while the infrared (IR) camera captured the infrared radiation from the specimen surface. The average value of the temperature was calculated with a high accuracy (0.02 K) and provided as the temperature change of the specimen. 

**Figure 4:**
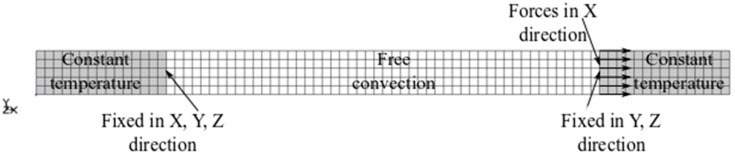
A mesh for the finite element model (FEM) of the SMA specimen showing loading and boundary conditions.

In order to simulate the real loading and boundary conditions of experiments, the finite element model (FEM), which consisted of 400 hexahedral 3D elements (80 × 5 × 1), was prepared. Due to the significant difference in volume and mass of the testing machine grips and specimen, the huge transient conduction was assumed. It was realized as a prescribed constant temperature at the specimens ends. The free convection on the specimen model was proposed between the testing machine grips. The mesh for the FEM of the SMA specimen with the loading and boundary conditions is presented in Figure 4. 

The loading process was simulated in 150 quasi-static time steps applying the tension forces on the one side, while the opposite side was fixed. According to the experiment, the loading force was kept constant for three minutes at various stages of the evident martensitic transformation, monitored by IR as localized (Programs 1, 2 and 3); see Section 2, Figure 2. 

**Figure 5:**
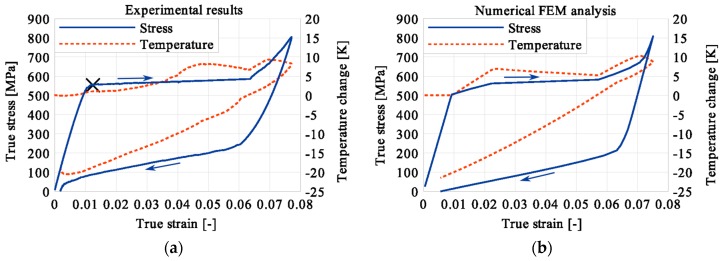
Stress and temperature variations vs. strain for the loading program (1): 3-min loading interruption at the beginning of the localized martensitic transformation (LMT): (**a**) experimental; (**b**) numerical results.

**Figure 6:**
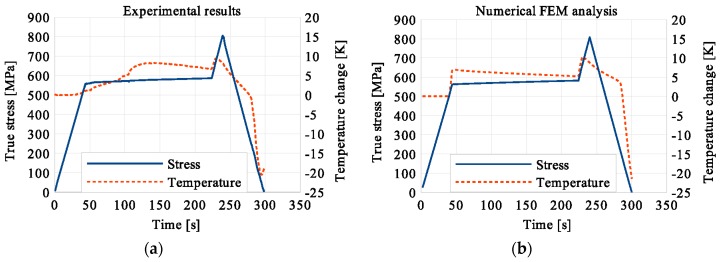
Stress and temperature variations vs. time for the loading program (1): 3-min loading interruption at the beginning of LMT: (**a**) experimental; (**b**) numerical results.

**Figure 7:**
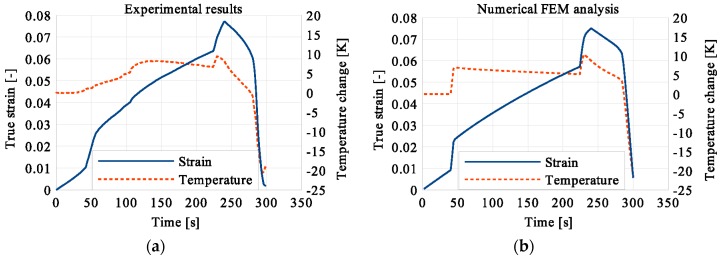
Strain and temperature variations vs. time for the loading program (1): 3-min loading interruption at the beginning of the LMT: (**a**) experimental; (**b**) numerical results.

**Figure 8:**
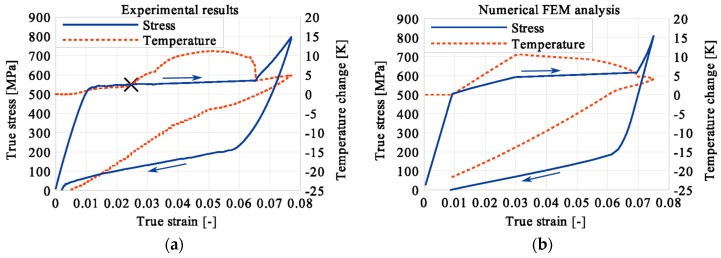
Stress and temperature variations vs. strain for the loading program (2): 3-min loading interruption in the middle of the LMT: (**a**) experimental; (**b**) numerical results.

**Figure 9:**
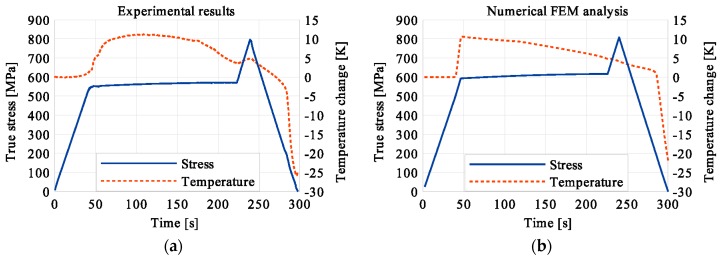
Stress and temperature variations vs. time for the loading program (2): 3-min loading interruption in the middle of the LMT: (**a**) experimental; (**b**) numerical results.

**Figure 10:**
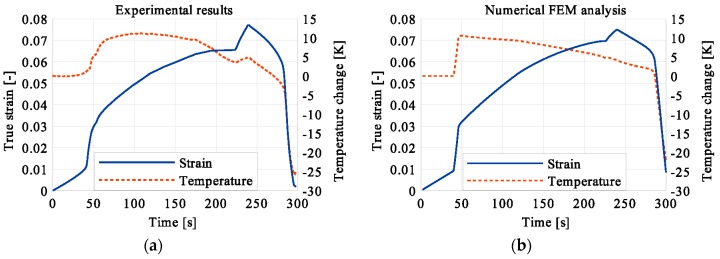
Strain and temperature variations vs. time for the loading program (2): 3-min loading interruption in the middle of the LMT: (**a**) experimental data; (**b**) numerical results.

**Figure 11:**
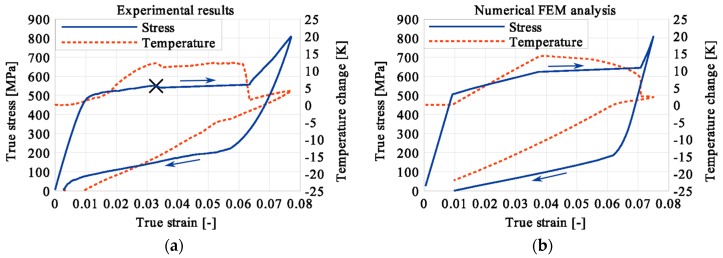
Stress and temperature variations vs. strain for the loading program (3): 3-min loading interruption at the end of the LMT: (**a**) experimental; (**b**) numerical results.

**Figure 12:**
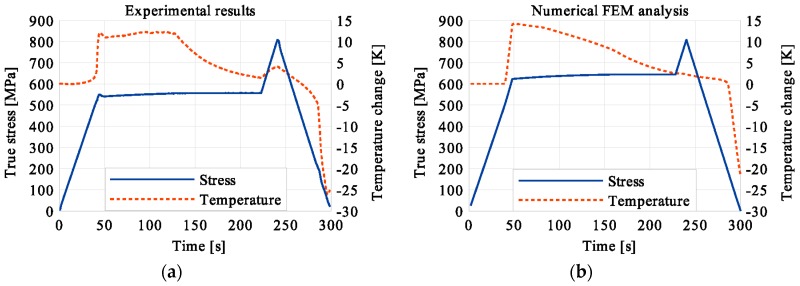
Stress and temperature variations vs. time for the loading program (3): 3-min loading interruption at the end of the LMT: (**a**) experimental; (**b**) numerical results.

**Figure 13:**
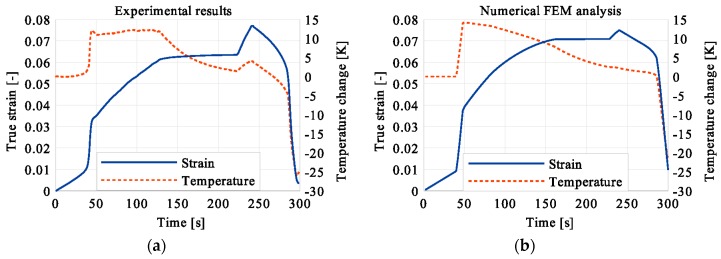
Strain and temperature variations vs. time for the loading program (3): 3-min loading interruption at the end of the LMT: (**a**) experimental; (**b**) numerical results.

In the period of loading interruption (loading force keeping constant), the specimen temperature decreased due to the free convection to the surroundings. A change of the temperature affected a further martensitic transformation. The martensitic volume fraction and the transformation strain continued increasing. The graphs presenting variations of stress and temperature vs. strain and stress, temperature, and strain vs. time are given in the consecutive Figure 5, Figure 6, Figure 7, Figure 8, Figure 9, Figure 10, Figure 11, Figure 12 and Figure 13 for all loading programs carried out.

The calibrated material parameters used for thermomechanical numerical analysis are given in Table 1.

Stress and the related temperature changes vs. strain characteristics obtained for the TiNi SMA subjected to the loading programs (1), (2), and (3) under a stress rate of 12.5 MPa s^−1^ at room temperature were presented in Figure 5, Figure 8 and Figure 11, whereas those of the stress and temperature changes vs. time in Figure 6, Figure 9 and Figure 12. In addition, the strain and temperature changes vs. time, which demonstrate an influence of the constant force period on the strain variation, were illustrated in Figure 7, Figure 10 and Figure 13. In all figures, the experimental data are presented on the left diagrams, whereas the corresponding numerical results on the right ones. The stages where the force was kept constant for three minutes are marked by **X** (Figure 5, Figure 8 and Figure 11).

One can notice in Figure 5, Figure 6 and Figure 7 that shortly after the start of martensitic transformation, the loading force was arrested and kept constant. A very short period of the stress-induced martensitic transformation before the loading pause gave a very small temperature increase of the material. This means that the martensitic phase transformation was at the very initial stage when the loading was interrupted. The small true stress increase in diagrams was related to the further strain development via the change of the cross-sectional area. As the loading continued, a significant increase of the specimen temperature was observed which confirmed that the stress-induced transformation process was in progress at this stage. 

Diagrams presented in Figure 8, Figure 9 and Figure 10 demonstrate that in the case of the program (2) where the period of constant loading was induced in the advanced stage of the LMT, the observed temperature increase, as the result of SIMT, was more significant. The temperature change can also be seen after the loading interruption. During the loading pause, there is latent heat production due to the exothermal phase transformation. As the temperature-induced phase transformation comes to an end, the production is smaller and the dissipation increases, which can be noticed easily on the diagrams. More importantly, it can be noticed in Figure 10 that at the end of the loading pause period the transformation was almost completed. After the loading continued, a much smaller temperature change is observed than in the program (1). The transformation-induced creep deformation potential is almost completely saturated and if the loading pause continues, the strain will not change anymore.

The diagrams presented in Figure 11, Figure 12 and Figure 13 demonstrate that in the case of the loading program (3), the SIMT before the loading pause produced the highest temperature increase compared to the programs (1) and (2). After the loading interruption at this deformation stage, the temperature-induced martensitic transformation was completed long before the end of the constant-force period. A transformation and total strains were constant from that stage (approximately middle of the pausing period) until the end of the loading pause. The end of the martensitic phase transformation was recognized by the faster temperature drop due to the convection to the environment and already completed phase transformation. After the loading was continued, only an insignificant increase of the temperature was observed.

## 5. Discussion

**Figure 14:**
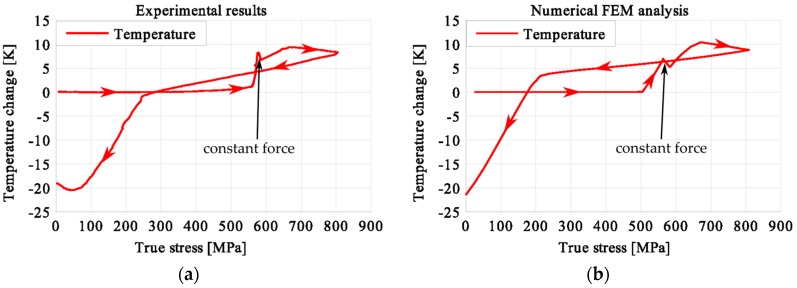
Temperature variations vs. stress for the loading program (1): 3-min loading interruption at the beginning of LMT: (**a**) experimental; (**b**) numerical results.

**Figure 15:**
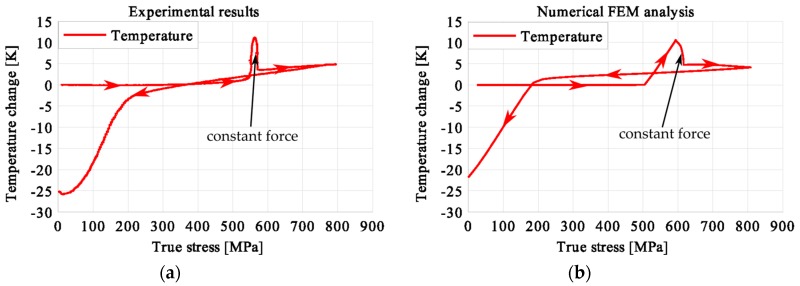
Temperature variations vs. stress for the loading program (2): 3-min loading interruption at the beginning of the LMT: (**a**) experimental; (**b**) numerical results.

**Figure 16:**
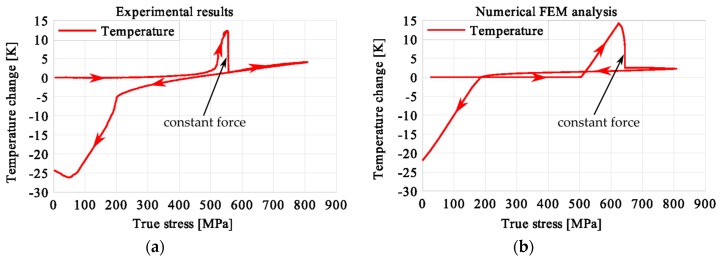
Temperature variations vs. stress for the loading program (3): 3-min loading interruption at the beginning of the LMT: (**a**) experimental; (**b**) numerical results.

The temperature variations vs. stress for three types of the loading program (1), (2) and (3) were depicted for experimental (left) and numerical (right) results in Figure 14, Figure 15 and Figure 16. The stage of constant force was marked in each figure. The temperature change during that period reveals an influence of the stress arrest during the phase transformation. 

Taking into account the results presented in Figure 5, Figure 8 and Figure 11 one can notice that the stress–strain curves are quite similar for all loading programs (1)-(3) carried out. The corresponding temperature and strain variations are much more diversified. Looking on the results presented in Figure 8, Figure 9 and Figure 10 for example, showing temperature variations captured during experiments and calculations carried out using the model, one can say that qualitatively they agreed reasonably well themselves. However, from the quantitative point of view some differences can be indicated. The main reason for such a situation results from the fact that the model uses 16 material parameters and their calibration still requires some additional work with respect to their adequate optimization. The most difficult task in this matter concerns the heat-transfer parameters like the convection or conduction coefficients, for example. To solve this problem effectively, more experimental data is required. On the other hand, the scatter between experimental and predicted values of temperature may be caused by the model feature assuming a homogeneity of the phase transformation and its omission in the elastic range. The experimental findings show however, that the phase transformation starts just before the end of elastic range, and the fields of martensitic transformation and temperature variation are not entirely homogeneous. These aspects of the paper require further thorough analyses.

It was illustrated in Figure 7, Figure 10, Figure 13, Figure 14, Figure 15 and Figure 16, that the transformation develops also when the specimen loading was kept constant. However, in such conditions the transformation process does not develop as fast as during the typical loading programs. The higher the strain reached before the loading interruption, the more the parent phase was transformed into the martensite. This follows the lower temperature increase recorded during the reloading, caused by the smaller amount of parent phase that was not transformed before. Furthermore, during the three minutes loading interruption, the temperature-induced transformation was developing, mainly due to the temperature change caused by convection. So, in the case of program (3), less of the parent phase was transformed into the martensite phase, since the transformation process was almost completed before and during the loading pause.

This is demonstrated either in the stress-strain curves obtained for various loading programs, Figure 5, Figure 8 and Figure 11 or temperature changes shown in Figure 14, Figure 15 and Figure 16. All the results are compared in Figure 17 in the case of experimental data and in Figure 18 for numerical calculations. 

The maximum transformation induced creep strain is related to the maximum transformation strain of the SMA specimen.

**Figure 19:**
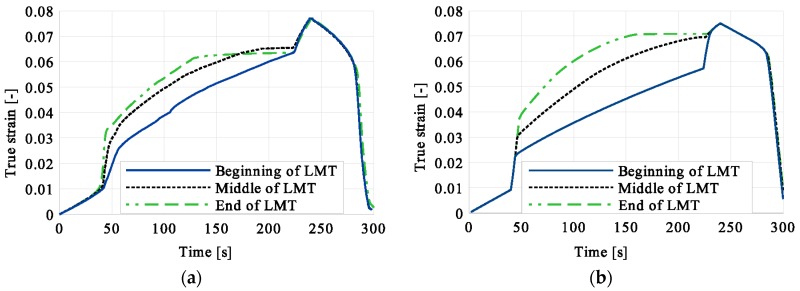
Strain vs. time comparison of experimental and numerical results for the loading program (3): 3-min loading interruption at the beginning of LMT: (**a**) experimental; (**b**) numerical results.

Finally, a comparison of the strain vs. time curves shown in Figure 7, Figure 10 and Figure 13 was presented in Figure 19: on the left-experimental, on the right-modeling results.

The strain vs. time curves presented in Figure 19 illustrate clearly that the moment of loading pause has a significant influence on the SMA mechanical/structure behavior.

## 6. Conclusions

Experimental and numerical results were presented for the TiNi SMA subjected to a modified program of the force-controlled tension, while the loading force was kept constant at the beginning, in the middle and at the end of the localized, stress-induced martensitic transformation. 

Development of the martensitic transformations in the SMA under such conditions was studied using a novel testing machine and fast and sensitive infrared camera. The temperature changes provide knowledge of how far the transformation is advanced and how it develops.

It was found that a pause in the loading process produced a further development of the transformation and total strain increase. The strain increase under constant loading conditions was recognized as the transformation-induced creep. 

It was confirmed by both experiment and model that thermo-mechanical coupling introduces significant influence on the transformation-induced creep phenomena in the TiNi SMA. Measuring temperature variations, accompanying the process, led to much better recognition of the phase transformation in SMA than the mechanical characteristics. 

The lower temperature increase, observed during the reloading after the loading pause, pointed on the higher saturation of the martensitic transformation during the pause. 

The creep-like effect observed during the periods of constant loading was well reproduced by the proposed constitutive model and partitioned thermomechanical coupling approach for all three loading programs carried out. 

Comparison of the results presented for various loading programs exhibited that the partitioned coupling approach of FEM software for the structural analysis PAKS and the heat transfer analysis PAKT describe properly the stress, strain and temperature variations obtained during the SMA loading program. 

## Figures and Tables

**Figure 17:**
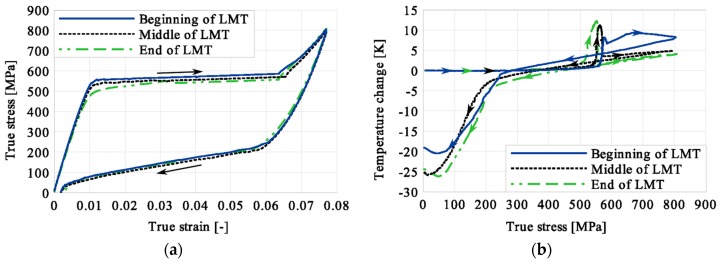
Comparison of experimental data obtained for TiNi SMA tension with 3-min loading interruption induced at the beginning (Program 1), middle (Program 2) and end (Program 3) of LMT: (**a**) stress vs. strain; (**b**) temperature change vs. stress.

**Figure 18:**
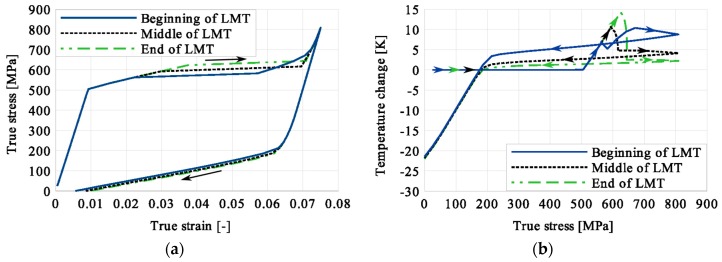
Comparison of numerical results obtained for TiNi SMA tension with 3-min loading interruption induced at the beginning (Program 1), middle (Program 2) and end (Program 3) of the LMT: (**a**) stress vs. strain; (**b**) temperature change vs. stress.

**Table 1:** TiNi SMA mechanical parameters used for thermo-mechanical numerical analysis.

Material Parameter	Value	Material Parameter	Value
*E_A_* (GPa)	53.2	ν (-)	0.41
*E_M_* (GPa)	50.9	*h* (W m^−2^ K^−1^)	6.5
α_A,M_ (K^−1^)	1.1 × 10^−5^	$c_{p}$ (J kg^−1^ K^−1^)	460
*H* (-)	0.066	$\lambda_{c}$ (W m^−1^ K^−1^)	18
$\rho \Delta s_{0}^{\left(A,M\right)}$ (MPa K^−1^)	−0.378	ρ (g cm^−3^)	6.29
$M_{0s}$ (K)	213	$M_{0f}$ (K)	209
$A_{0s}$ (K)	270	$A_{0f}$ (K)	276
